# Classification of mild Parkinson’s disease: data augmentation of time-series gait data obtained via inertial measurement units

**DOI:** 10.1038/s41598-023-39862-4

**Published:** 2023-08-03

**Authors:** Hirotaka Uchitomi, Xianwen Ming, Changyu Zhao, Taiki Ogata, Yoshihiro Miyake

**Affiliations:** 1 Department of Computer Science, School of Computing, Tokyo Institute of Technology, Yokohama, 226-8502 Japan; 2 Department of Systems and Control Engineering, School of Engineering, Tokyo Institute of Technology, Yokohama, 226-8502 Japan

**Keywords:** Computer science, Parkinson's disease, Physical examination

## Abstract

Data-augmentation methods have emerged as a viable approach for improving the state-of-the-art performances for classifying mild Parkinson’s disease using deep learning with time-series data from an inertial measurement unit, considering the limited amount of training datasets available in the medical field. This study investigated effective data-augmentation methods to classify mild Parkinson’s disease and healthy participants with deep learning using a time-series gait dataset recorded via a shank-worn inertial measurement unit. Four magnitude-domain-transformation and three time-domain-transformation data-augmentation methods, and four methods involving mixtures of the aforementioned methods were applied to a representative convolutional neural network for the classification, and their performances were compared. In terms of data-augmentation, compared with baseline classification accuracy without data-augmentation, the magnitude-domain transformation performed better than the time-domain transformation and mixed-data augmentation. In the magnitude-domain transformation, the rotation method significantly contributed to the best performance improvement, yielding accuracy and F1-score improvements of 5.5 and 5.9%, respectively. The augmented data could be varied while maintaining the features of the time-series data obtained via the sensor for detecting mild Parkinson’s in gait; this data attribute may have caused the aforementioned trend. Notably, the selection of appropriate data extensions will help improve the classification performance for mild Parkinson’s disease.

## Introduction

State-of-the-art technologies are applied for gait analysis, which allow for the early detection and diagnostic assistance for Parkinson's disease (PD) and other neurological intractable diseases. PD is a long-term neurodegenerative disorder of the central nervous system that causes gait disorders^1–4^. In clinical practice, PD diagnosis is completely based on neurological examinations, which focus on the observation of motor signs, and rating scales, such as the unified Parkinson’s disorders rating scale (UPDRS) and/or the Hoehn and Yahr scales (H&Y)^5,6^. Furthermore, considering the importance of determining minor symptoms that appear over longer periods of time than those needed for a clinical examination^7,8^, state-of-the-art technologies must investigate the detection of PD gait by measuring and analyzing human gait movements. In this regard, through wearable sensors, such as inertial measurement units (IMUs), and a video camera, gait information can be effectively recorded. Considering gait measurement in various clinical environments, the IMU-based method is a more cost-efficient and convenient method than the video camera-based method; the IMU-based method is less likely to cause problems with relatively long-term measurement difficulties that are caused by variations of structural noise (occlusion and illumination), clutter, appearance alternations owing to changes in the 3D viewpoint, and other perceptual distortions^9–11^.

In recent IMU-based implementations for classifying gait information, not only machine learning methods^12,13^ but also deep learning methods^14,15^ have been investigated. In particular, a deep learning method automatically learns features concerning the correspondence between input and output data using large datasets. Dehzangi et al. devised a deep convolutional neural network (CNN) with gait data obtained from multiple IMUs to identify individuals; five IMU sensors were attached at the chest, lower back, right-hand wrist, right knee, and right ankle positions of each participant^14^. Moreover, Nguyen et al. proposed an IMU-based spectrogram approach with CNN for gait classification for detecting abnormal gait corresponding to musculoskeletal disease. The gait-spectrogram dataset was generated based on time–frequency analysis^15^. This approach involved data from seven IMU sensors placed on seven body positions of 69 individuals comprising healthy participants, athletes, and participants with foot abnormalities, walking at multiple speeds. Reportedly, the subject groups could be predicted with data from only a single IMU sensor at the pelvis position, with an accuracy of 97.58%. Although various studies have focused on abnormal gait classification using deep learning with IMU data, detection of gait signature indicating brain- and nerve-system disorders in early stages, such as mild PD, and classification of mild PD and healthy subjects remain unexplored from the viewpoint of deep learning. Moreover, training a deep learning network generally requires a large and diverged dataset encompassing a substantial number of data instances that may occur in practice^16,17^. In concrete, this kind of detection of mild PD gait disorder has two distinctive problems. First, the data size is small because the dataset is from the clinical domain. The symptoms are not obvious at the beginning of the disease and are easily ignored. Most patients in hospitals have severe PD, and very few have mild PD. Secondly. The data are difficult to distinguish. Data from prior studies, for example, were captured from normal people, athletes, and patients^15^, which can be easily distinguished. However, mild PD of normal people is highly similar to that of elderly. Distinguishing these may be difficult for doctors without careful assessment. To address this issue, data augmentation is needed.

Data augmentation improves the performance and results of machine learning models by training datasets based on newly formulated and unique examples. If the dataset in a machine learning model is rich and sufficient, then the model’s performance and accuracy will greatly improve. Data augmentation is an important technique that involves applying transformations to training data to increase their quantity and quality. Data augmentation is used for two primary reasons: (1) to increase the amount of data available for training and (2) to prevent overfitting. Deep learning models have become popular in recent years because of their ability to achieve high classification accuracy. However, these models often contain many layers with numerous parameters, which can require large amounts of data to learn effectively. In some cases, obtaining sufficient data may be difficult, especially when dealing with sensitive data, such as medical records. Data augmentation can help overcome this issue by enabling models to learn from a small amount of data. Overfitting occurs when a model fits the training data exceedingly well, resulting in poor performance when tested on new data. The primary goal of creating a deep learning model is to accurately classify and evaluate new data. Therefore, ensuring that the model is not biased toward the training data is crucial. Data augmentation techniques can help prevent overfitting by creating new variations of the training data that the model has not seen before. In summary, data augmentation is a crucial technique in deep learning that helps increase the quantity and quality of training data and prevents overfitting. By appropriately augmenting the training data, deep learning models can be trained to generalize well and achieve high performance on new data.

Reportedly, the representative demonstrations showing the effectiveness of data augmentations are in the field of transformations on computer vision, such as horizontal flipping, color-space augmentations, and random cropping. Most original proposals of the state-of-the-art CNN^9^ architectures have utilized a certain mode of data augmentation. Although data augmentation is a commonly used method for neural network-based image recognition, it is yet to be established as a standard procedure for time-series pattern recognition^18^. As reported in the literature, in data obtained via wearable IMU sensors, data augmentation can address unexplored input space, prevent overfitting, and improve the generalization ability of a deep learning model^19^. The need for generalization is especially important for real-world data and can help networks overcome limitations involving small datasets. Similar to data augmentation for images, most data augmentation techniques for time-series data are based on random transformations of the training data. In this regard, random noise^20^, cropping^21^, scaling^22^, random warping in the time dimension^20,22^, and frequency warping^23^ have been introduced in data recorded via wrist-mounted IMU and other natural time-series data without gait information. Notably, certain challenges are associated with random transformation-based data augmentation: a diverse volume of time-series data is present, with each time-series possessing different properties, and not every transformation is applicable to every dataset^22^. For example, jittering (adding noise) assumes the time-series patterns of the particular dataset to be noisy. While this aspect might be true for sensor, audio, or electroencephalogram data, it is not necessarily true for time-series data based on object contours, such as those from the IMU sensors. Moreover, data augmentation methods are task-dependent, and data augmentation methods that are effective for human-activity recognition might not be effective for mild PD classification because the data exhibit different features and properties^24^. Thus, it is not sufficiently clear what type of data augmentation for IMU gait data is important in the method of based on the IMU gait data, deep learning is utilized for detecting mild PD through the classification of mild PD and healthy subjects, and the appropriate type of data augmentation for this data remains unidentified. More specifically, previous study most related to the task of detecting mild PD based on such IMU gait data was^20^, which used a wrist IMU to monitor the motor status of PD patients while using data augmentation to address small dataset sizes, noisy labels, and large intraclass variability. Although some challenges, such as small data sizes, matched those of the task, the core challenge of our study, which is the high similarity between mild PD and healthy elderly, did not appear in^20^. It is unclear whether the analysis method used in this previous study is effective in detecting mild PD based on IMU gait data, which is the focus of this study.

In the aforementioned studies, deep learning-based data augmentation for gait data with IMU has not been fully considered. Moreover, in relevant research concerning mild PD patients, the effective data-augmentation method for gait data has not been characterized. Furthermore, further improvements are needed in terms of the number of IMU sensors, and the classification accuracy requires further improvement. Therefore, in the current study, effective data-augmentation methods were investigated for deep learning to classify participants with mild PD compared with healthy participants (HPs). For the classification of mild-PD participants, a representative CNN-based deep learning model and candidates of data augmentation methods were employed to classify mild PD with time-series data obtained via an IMU. The data augmentation methods are rotation^25^, jittering^26^, scaling^12^, magnitude warping^20^, permutation^20^, cropping^27^, and time warping^20^. These data augmentation methods were considered in this study because these have been applied to time-series data for human-activity recognition; however, their effectiveness in mild PD detection remains unknown. To address the identified gaps in the literature, this study gathers and presents these data augmentation techniques, aiming to characterize effective data-augmentation methods for the classification of participants with mild PD and HP through deep learning.

## Methods

In this study, a representative CNN-based method was used as representative method to classify the age-matched mild PD and healthy control groups. Various data augmentation methods were applied to the model, and the effectiveness of the data augmentation methods was compared and verified.

This section presents three major subsections: representative CNN-based classification flowchart, data augmentation methods, and evaluation method. The subsection on data augmentation methods is further divided into magnitude-domain transformations (rotation, jittering, scaling, magnitude warping), time-domain transformations (permutation, time warping, cropping), and the mixed data-augmentation method featuring mixtures of these two transformations. The subsection on the evaluation method presents the experimental setup, dataset, evaluation index, and validation method.

### Representative CNN-based classification flowchart

**Figure 1:**
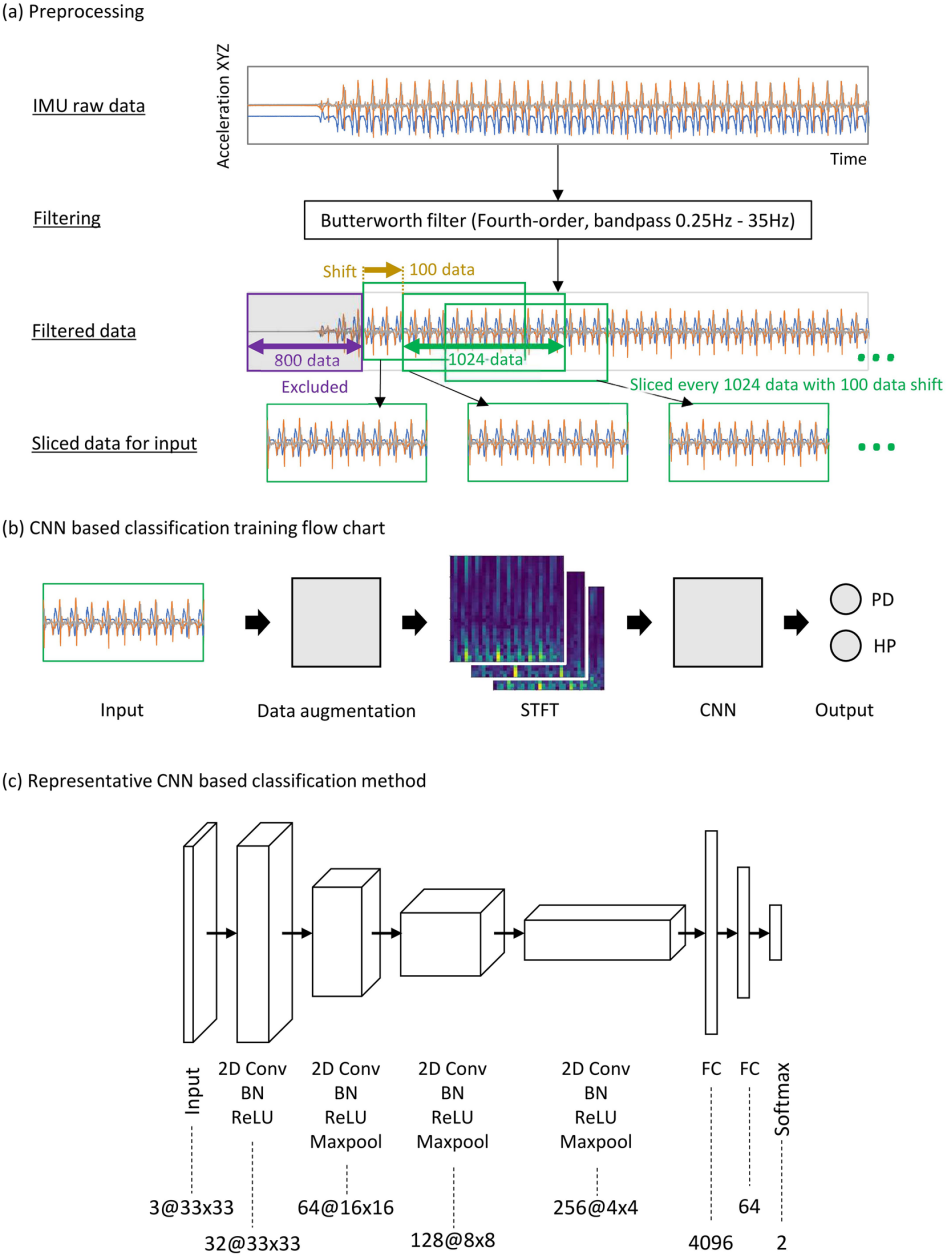
Data-processing pipeline used in this study. (**a**) Preprocessing of the input dataset for the CNN-based deep learning model comprises the filtering process using a fourth-order Butterworth filter (bandpass: 0.25–35 Hz) and the slicing process, which sliced the measured raw time-series data into 1024 slices of uniform data length. (**b**) The training flowchart for the CNN-based classification sequentially consists of the input data obtained via the slicing process, specific data augmentation, short-time Fourier transform (STFT), and the representative CNN-based classification model. (**c**) The representative CNN-based classification model is structured using 2D convolution layers with batch normalization (BN), the Rectified Linear Unit (ReLU), and maxpooling layers (Maxpool), outputting the parameters of the Softmax function for the mild Parkinson’s disease patient and healthy elderly participant.

Figure 1 illustrates the preprocessing and structure of a representative CNN-based classification model. After recording the acceleration data via IMU sensors, normalization and slicing are performed such that the data length is adjusted to 1024 data in x-, y-, and z-axes with 100 data shifts, as depicted in Fig. 1a. After filtering the data through a bandpass filter, data augmentation methods are applied to the filtered data, as demonstrated in Fig. 1b. Subsequently, the augmented data are utilized as the input of the CNN model, as depicted in Fig. 1c.

For preprocessing the acceleration data, a bandpass filter is applied to the accelerometer data to eliminate the gravity-induced DC component as well high-frequency noise^28^. Based on a series of experiments, a fourth-order Butterworth bandpass filter with a lower and higher cut-off frequencies of 0.25 and 35 Hz is used^29^. Characteristically, in the Butterworth filter, the frequency–response curve in the passband is maximally flat.

After applying the fourth-order Butterworth bandpass filter, a spectrum is calculated for the filtered acceleration data. A spectrogram is used to represent the time–frequency distribution of signals as the inputs of deep CNN models. A spectrogram can be generated from a time-domain signal by applying the Fourier transform^9^. Spectrograms represent sequences of a spectrum, with time and frequency represented on the axes and the brightness, which indicates the strength of a frequency component at each interval. In particular, a time-domain signal solely represents a signal's amplitude trends, and spectrograms are more visually recognized and intelligible patterns. In this research, combinations of spectro-temporal representations transformed from raw IMU-recorded data were applied to the CNN model for classification.

After calculating the spectrum, the calculated spectrum was used by the CNN to classify the mild PD and healthy elderly. In this research, a representative CNN-based classification model is defined and tested to evaluate the effectiveness of various data augmentation methods. The main functional elements of the CNN model are as follows: two-dimensional convolutional layers (2D Conv), batch normalization layers (BN), activation layers using Rectified Linear Unit (ReLU), and maxpooling layers (Maxpool). The representative CNN model featured in this study can be visualized as illustrated in Fig. 1c. The CNN was trained using the following parameters: 300 epochs, batch size of 64, and Adam optimizer. The learning rate was set to 0.0001, and the weight decay to 0.000001.

### Data augmentation methods

To investigate appropriate data augmentation methods for the mild PD classification framework based on IMU-collected gait data, this study exhaustively investigated and compared a variety of data augmentation methods. Magnitude- and time-domain transformations were used for data augmentation. In addition, as a supplemental investigation, a combination of these transformations was applied through data augmentation methods.

### Magnitude-domain transformation

In magnitude-domain-transformation-based data augmentation, transformation is performed on the values of the time-series. As an important characteristic of magnitude transformations, only the values of each element are modified and the time-steps are kept constant. This magnitude-domain-transformation-based data augmentation was subdivided into the following types: rotation, jittering, scaling, and magnitude-warping methods.

#### Rotation method

The input-to-output conversion, based on the rotation method, is defined as

1 $$\begin{array}{*{20}c} {\user2{x^{\prime}}_{{\varvec{t}}} = {\varvec{R}}_{{\varvec{n}}} \left( \theta \right){\varvec{x}}_{{\varvec{t}}} , \left( {t = 1,2,3, \ldots T} \right)} \\ \end{array}$$

where ${\varvec{x}}_{{\varvec{t}}} = \left( {x_{t} , y_{t} , z_{t} } \right)^{T}$ symbolizes the input data, $\user2{x^{\prime}}_{{\varvec{t}}} = \left( {x^{\prime}_{t} , y^{\prime}_{t} , z^{\prime}_{t} } \right)^{T}$ indicates the output data of this method, $t$ denotes the time-stamp, $T$ depicts the length of input data, ${\varvec{R}}_{{\varvec{n}}} \left( \theta \right)$ symbolizes the representative matrix of Rodrigues' rotation formula, ${\varvec{n}} = \left( {n_{x} , n_{y} , n_{z} } \right)^{T}$ indicates an axial unit vector of any rotation axis passing through the origin of the coordinates in 3D space, and ${\varvec{\theta}}$ denotes the rotation angle of ${\varvec{x}}_{{\varvec{t}}}$ around the rotation axis ${\varvec{n}}$.

The representative matrix of Rodrigues' rotation formula, ${\varvec{R}}_{{\varvec{n}}} \left( \theta \right)$, is given as

2 $$\begin{array}{*{20}c} {{\varvec{R}}_{{\varvec{n}}} \left( \theta \right) = \left( {\begin{array}{*{20}l} {n_{x}^{2} \left( {1 - \cos \theta } \right) + \cos \theta } \hfill & {\quad n_{x} n_{y} \left( {1 - \cos \theta } \right) - n_{z} \sin \theta } \hfill & {\quad n_{x} n_{z} \left( {1 - \cos \theta } \right) - n_{y} \sin \theta } \hfill \\ {n_{x} n_{y} \left( {1 - \cos \theta } \right) - n_{z} \sin \theta } \hfill & {\quad n_{y}^{2} \left( {1 - \cos \theta } \right) + \cos \theta } \hfill & {\quad n_{y} n_{z} \left( {1 - \cos \theta } \right) - n_{x} \sin \theta } \hfill \\ {n_{x} n_{z} \left( {1 - \cos \theta } \right) - n_{y} \sin \theta } \hfill & {\quad n_{y} n_{z} \left( {1 - \cos \theta } \right) - n_{x} \sin \theta } \hfill & {\quad n_{z}^{2} \left( {1 - \cos \theta } \right) + \cos \theta } \hfill \\ \end{array} } \right)} \\ \end{array}$$

**Figure 2:**
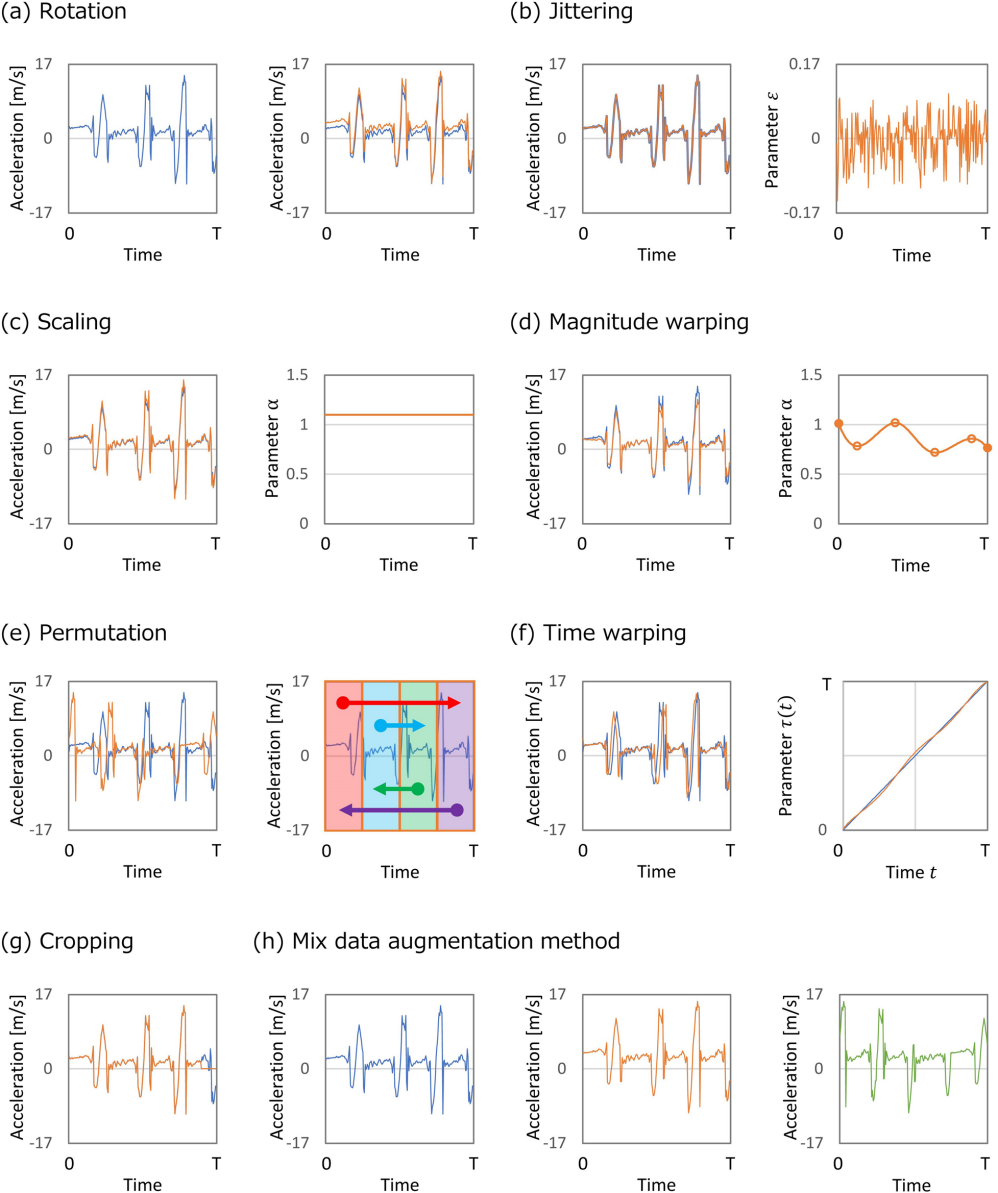
Examples of data augmentation methods explored in this study. The blue line shows the original time-series not converted via data augmentation. The orange line indicates the converted time-series based on a specific data augmentation method as follows: (**a**) Left graph with a blue line presents the original time-series of shank-worn IMU acceleration in a human-gait axis. Right graph with an orange line shows the time-series converted based on the rotation method in data augmentation, with the blue line depicting the original time-series, allowing a result comparison before and after augmentation. (**b**) Left graph shows the converted time-series based on the jittering method. Right graph plots the time-series of noise added to the original time-series. (**c**) Left graph shows the converted time-series based on the scaling method. Right graph illustrates the change over time in the static scaling-parameter value as an example. (**d**) Left graph displays the converted time-series based on the magnitude warping method. Right graph shows the change over time in the value of a dynamic scaling parameter as an example. (**e**) Left graph depicts the converted time-series based on the permutation method. Right graph presents the segmentation parts and exchanging relationship among the segmented parts. (**f**) Left graph shows the converted time-series based on the time-warping method. In the right graph, the orange line indicates the value of a dynamic scaling parameter of the time-warping method compared with the timestamp of the original time-series shown as the blue straight line. (**g**) Left graph shows the converted time-series based on the cropping method. The last 10% length of time-series was excluded. (**h**) An example of a mixed data-augmentation method using the rotation and permutation methods. Left graph plots the original time-series. The center graph shows the converted time-series obtained via the rotation method from the original time-series. Right graph depicts the time-series converted via the permutation method for the time-series converted through the rotation method. This research experimentally investigated the following four types of mixed data-augmentation methods: combinations of rotation–scaling, rotation–jittering, jittering–permutation, and rotation–scaling–magnitude warping, respectively.

In this research, the rotation axis (${\varvec{n}}$**)** and rotation angle (${\varvec{\theta}}$**)** were probabilistically determined based on a uniform distribution. The range of rotation angles (${\varvec{\theta}}$**)** ranged from − 15° to 15°, in accordance with the literature^25^. In Fig. 2a, the left graph plotted in blue features the original data of an axis before data augmentation; the right graph plotted in blue presents the data augmented using the rotation method compared with the original data (blue graph).

#### Jittering method

The jittering method is an approach for simulating additive sensor noise. Jittering is expressed as follows:

3 $$\begin{array}{*{20}c} {x^{\prime}_{t} = x_{t} + \varepsilon_{t} , \left( {t = 1,2,3, \ldots T} \right)} \\ \end{array}$$

where ${\varvec{x}}_{{\varvec{t}}} = \left( {x_{t} , y_{t} , z_{t} } \right)^{T}$ represents the input data, and $\user2{x^{\prime}}_{{\varvec{t}}} = \left( {x^{\prime}_{t} , y^{\prime}_{t} , z^{\prime}_{t} } \right)^{T}$ depicts the output data of this method. Equation (3) relates to $x^{\prime}_{t}$ as well as to $y^{\prime}_{t}$ and $z^{\prime}_{t}$. $\varepsilon$ depicts the Gaussian noise added to each time-step ($t$); $\varepsilon \sim N\left( {0, \sigma^{2} } \right)$. The standard deviation ($\sigma$) of the added noise is a hyperparameter that must be pre-determined. In this research, $\sigma = 0.1$, in accordance with the literature^26^. A commonly used method—the addition of noise to the inputs—is beneficial for increasing the generalization of neural networks. Through this approach, the generalization is improved by effectively creating new patterns with the assumption that the unseen test patterns solely differ from the training patterns by a factor of noise. In addition, jittering is demonstrably advantageous for mitigating time-series drift in various neural network models^30^. Time-series drift occurs when the data distribution changes owing to the introduction of new data. The left graph presented in Fig. 2b shows the original data of an axis before data augmentation as the blue line and augmented data via the jittering method as the orange line. The right graph in Fig. 2b illustrates the noise ($\varepsilon )$ added to the original data.

#### Scaling method

The scaling method alters the global magnitude or intensity of a time-series by a random scalar value. For a scaling parameter of α, scaling is defined as a multiplication of α with the entire time-series, which is formulated as

4 $$\begin{array}{*{20}c} {x^{\prime}_{t} = \alpha x_{t} , \left( {t = 1,2,3, \ldots T} \right)} \\ \end{array}$$

The scaling parameter $\alpha$ can be determined by a Gaussian distribution $\alpha \sim N\left( {1, \sigma^{2} } \right)$ with $\sigma$ as a hyperparameter, and, in this study, $\sigma = 0.2$, referring to the available literature^12^. Notably, *scaling* in terms of time-series is different than that in the image domain. For time-series, *scaling* refers to simply increasing the magnitude of the elements while ensuring that the time-series is not enlarged. The left graph of Fig. 2c shows the original data of an axis before data augmentation as the blue line and data augmented via the scaling method as the orange line. The right graph in Fig. 2c presents the scaling parameter ($\alpha$) in this method.

#### Magnitude warping method

The magnitude warping method^20^ is a time-series specific data augmentation technique that warps a signal’s magnitude through a smoothed curve. Particularly, augmented time series is represented as

5 $$\begin{array}{*{20}c} {x^{\prime}_{t} = \alpha_{t} x_{t} , \left( {t = 1,2,3, \ldots T} \right)} \\ \end{array}$$

where the scaling parameter, $\alpha_{t} ,$ is a sequence created by interpolating a cubic spline, $S_{i} \left( u \right) = a_{i} + b_{i} \left( {u - u_{i} } \right) + c_{i} \left( {u - u_{i} } \right)^{2} + d_{i} \left( {u - u_{i} } \right)^{3} , \left( {u_{i} \le u \le u_{i + 1} } \right),{\text{ with}}\;{\text{knots}} \;u_{1} , \ldots ,u_{i} , \ldots ,u_{I} .{ }$ Each knot, $u_{i}$, is assumed from a Gaussian distribution $u_{i} \sim N\left( {1, \sigma^{2} } \right)$, where the number of knots ($I$) and standard deviation ($\sigma$) denote hyperparameters. In the present study, $\sigma = 0.2$ and $I = 4$, which were set in accordance with findings reported in the literature^20^. Herein, the initial and final $\alpha_{t}$ values are given by $N\left( {1, \sigma^{2} } \right)$. In magnitude warping, minor fluctuations in the data are added by increasing or decreasing random regions in the time-series. Notably, in magnitude warping for data augmentation, the random transformation is assumed to be realistic, and this augmentation depends on two pre-defined hyperparameters (the number of knots, $I$, and standard deviation of the knot height, $\sigma$), whereas other transformation-based methods are based on a single hyperparameter. In the left graph of Fig. 2d, the original data of an axis (before data augmentation) are depicted as the blue line, and data augmented by the magnitude warping method are depicted as the orange line. The right graph in Fig. 2d presents the scaling parameter ($\alpha_{t}$) used in this method. Herein, the four orange dots (white background) represent randomly set knots, and the two solid orange dots indicate randomly set starting and end points.

### Time-domain transformation

In time-domain-transformation-based data augmentation, the elements of the time-series are displaced to time-steps different than those of the original sequence. An important characteristic of time transformations is that the time-series characteristics of the time-series data change intrinsically. Time-domain-transformation-based data augmentations are categorized as permutation, time-warping, and cropping methods.

#### Permutation method

The permutation method for data augmentation was proposed by Um et al.^20^ for rearranging segments of a time-series, thereby generating a new pattern. Permutation is a convenient approach for randomly perturbing the temporal location of within-window events. To perturb the location of the data in a single window, the data are initially sliced into N segments having the same length, where N ranges from 1 to 5, and the segments are randomly permutated to construct a new window. Here, a value of N = 1 indicates that the segmented data frame is simply replicated. On each data-augmentation occasion, N was randomly determined from 1 to 5.

The left graph in Fig. 2e visualizes the original data of an axis before data augmentation as the blue line and the augmented data as the orange line with the permutation method. The right graph in Fig. 2e shows the data segmentation and replacement of segmented data.

#### Time warping method

The time warping^20^ is the act of perturbing a pattern in the temporal dimension, smoothly distorting the time intervals between samples and changing the temporal locations of the samples. When using time warping with a smooth warping path, the augmented time-series become

6 $$\begin{array}{*{20}c} {x^{\prime}_{t} = x_{\tau \left( t \right)} , \left( {t = 1,2,3, \ldots T} \right)} \\ \end{array}$$

where $\tau \left( t \right)$ is a warping function that warps the time steps based on a smooth curve. The smooth curve $\tau \left( t \right)$ is created by a cubic spline $S_{i} \left( u \right) = a_{i} + b_{i} \left( {u - u_{i} } \right) + c_{i} \left( {u - u_{i} } \right)^{2} + d_{i} \left( {u - u_{i} } \right)^{3} , \left( {u_{i} \le u \le u_{i + 1} } \right)$ with knots $u_{1} , \ldots ,u_{i} , \ldots ,u_{I}$. The parameter $I$ means the number of knots, and in this research, $I = 4$. The height of the knots, $u_{i} ,$ is obtained from $u_{i} \sim N\left( {1, \sigma^{2} } \right)$. Thus, the time steps of the series feature a smooth transition between stretches and contractions. The left graphs in Fig. 2f show the original data of an axis before data augmentation as the blue line and augmented data with the time warping method as the orange line. The right graph in Fig. 2f presents the warping function, $\tau \left( t \right)$, as the orange line, and the original data of the baseline as the blue line.

#### Cropping method

The cropping method is similar to image cropping or window slicing^27^ and is applied for diminishing the dependency on event locations by cropping 10% information. Specifically, this cropping method was implemented by replacing the last 10% of the target data with zeros. In Fig. 2g, the original data of an axis before data augmentation is presented as the blue line, and the data augmented with the cropping method is plotted as the orange line.

### Mixed data-augmentation method

This study primarily evaluated the effectiveness of individual data augmentation methods based on the above description. Specifically, magnitude-and time-domain transformations were used. Additionally, a combination of these transformations was applied through data augmentation methods as a supplemental investigation for further discussion, after the evaluation of the individual respective data augmentations. Combinations of various data augmentation methods have been used for multiple time-based transformation of data through the individual method^20^. For example, when using rotation–scaling combination, the rotation method and the scaling method were applied sequentially to the same dataset. In Fig. 2h, the left graph shows the original data of an axis before data augmentation, the center graph displays the augmented data obtained via the rotation method, and the right graph plots the augmented data obtained through the permutation method after the rotation method. This research experimentally examined the following four types of mixed data-augmentation methods: combinations of rotation–scaling, rotation–jittering, jittering–permutation, and rotation–scaling– magnitude warping, respectively.

### Evaluation method

To validate the performance of the proposed deep learning-based data augmentation, the experiments were conducted on a computer (Platform: Windows; graphics card: NVIDIA GeForce RTX 5000; architecture: CUDA 9.0). The construction of neural networks for the CNN was performed using torch 1.8.1 on Python 3.8. In the following subsections, the dataset, evaluation index, and validation method used in the experiments are described.

### Experimental participant and dataset

Forty-six mild PD patients, featuring a modified Hoehn & Yahr scale (mHY) of 1.0–2.0 (age: 68.7 ± 9.8 years, 22 male patients; 24 female patients), and 44 elderly healthy participants (age: 73.9 ± 6.0 years, 12 male participants and 32 female participants) participated in the experiment. This dataset was based on that from our previous study, featuring an expansion of the number of experimental participants^31^. The study was conducted in accordance with the Declaration of Helsinki. This experiment was approved by the Ethics Committee of the Tokyo Institute of Technology and the Ethics Committee of Kanto Central Hospital. Written informed consent was obtained from all participants. Data were collected via IMUs (TSND121, ATR-Promotions) to acquire the 3D accelerations at a sampling rate of 100 Hz. The experimental participants wore two IMUs on their left and right shanks. Data were collected as the participants walked at a general pace with the IMUs attached onto their left and right feet. Participants were instructed to walk back and forth in a straight corridor, with over 60 strides. The participants walked without any walking support. Data collected from the left and right IMUs were used together in this experiment without distinction. Therefore, 180 time-series of shank-worn IMU data were obtained in this study. The average data length was 10,066 ± 4232 at a sampling rate of 100 Hz (100.66 ± 42.32 s). Note that the first 8 s of all IMU gait data were excluded from the analysis for eliminating the initial unstable gait state. After slicing the time-series as shown in the classification flowchart in Fig. 1a, the total number of input data was 28,884. By applying each data augmentation method, the amount of training data effectively doubled after augmentation. Data augmentation was applied only to the training data and not to the test data.

### Evaluation index

To design the evaluation method in this research, four metrics were chosen to evaluate the classification result of PD patients and healthy people, as follows: accuracy, precision, recall, and F1-score. The detailed explanations for each metric are presented herein. First, the following four parameters were defined. True positive (TP) is an outcome wherein the model correctly predicts the positive class, which are the real PD-labeled data in the test dataset and are classified as PD by training. True negative (TN) is an outcome wherein the model correctly predicts the negative class, which are the real healthy people-labeled data in the test dataset and are classified as healthy people through training. False positive (FP) is an outcome wherein the model incorrectly predicts the positive class, which are the real PD-labeled data in the test dataset and are classified as healthy people via training. False negative (FN) is an outcome wherein the model incorrectly predicts the negative class, which are the real healthy people-labeled data in the test dataset and are classified as PD through training.

The accuracy represents the ratio of the correctly predicted number of samples to the total number of samples. Particularly, the accuracy is a feasible evaluation metric for classification problems, which are well-balanced and not skewed or feature no class imbalance. Therefore, the accuracy can be expressed as

7 $$Accuracy = \frac{TP + TN}{{TP + FP + FN + TN}}$$

The precision represents the proportion of positive identifications that are correct in practice, which is expressed as follows:

8 $$precision = \frac{TP}{{TP + FP}}$$

The recall is a measure of how many of the positive cases the classifier correctly predicted, over all the positive cases in the data. It is sometimes also referred to as sensitivity. It is expressed as follows:

9 $$recall = \frac{TP}{{TP + FN}}$$

The F1-Score is a measure encompassing the precision and recall. This combined measure is generally described as the harmonic mean of the two-constituent metrics. In essence, the harmonic mean is an alternative approach for calculating the *average* of values, which is more suitable for ratios (such as precision and recall) than the traditional arithmetic mean. The formula used for F1-score in this case is

10 $$F1 = 2 \times \frac{precision \times recall}{{precision + recall}}$$

The F1-score ensures a balance between the precision and recall for the classifier. Accordingly, this research mainly focused on the F1 score and accuracy.

### Validation method

The validation method is not adequately reliable because the accuracy obtained for one test set can vary considerably from the accuracy obtained for a different test-set. K-fold cross-validation addresses this problem by dividing data into folds and ensuring that each fold is used as a testing set at a certain point.

In this research, we utilized fivefold cross validation. Herein, the dataset is split into five folds. In the first iteration, the first fold is employed to test the model, and the remaining folds are used to train the model. In the second iteration, the second fold is leveraged as the testing set, whereas the remaining folds act as the training set. This process is repeated until each of the five folds is used as the testing set. This fivefold cross validation was conducted subject-independent, that is, different subjects were used in each fold.

## Result

**Figure 3:**
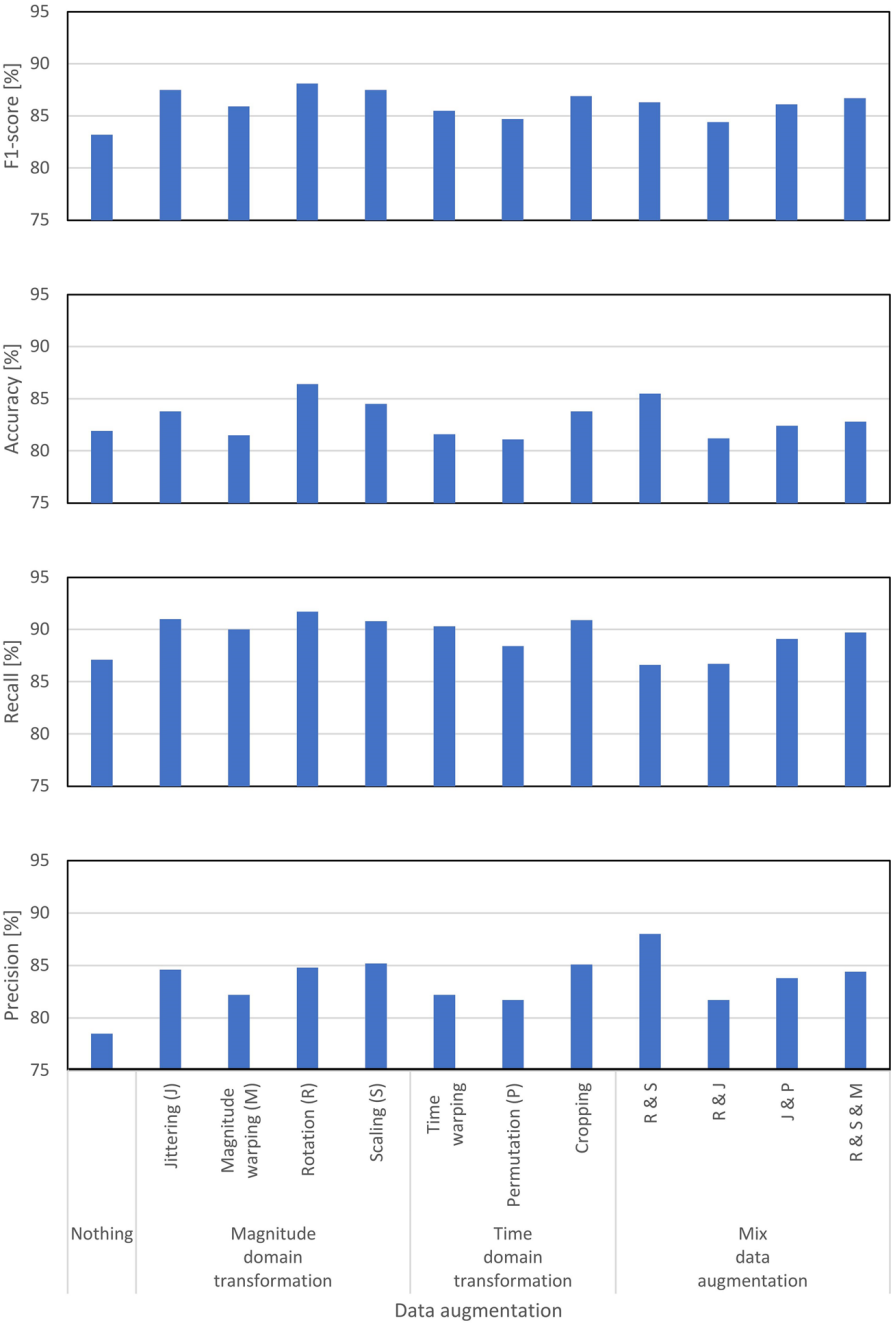
Performance evaluation results of data augmentation methods (bar-charts). The data augmentation methods explored are four methods of magnitude-domain transformation, including rotation (R), jittering (J), scaling (S), and magnitude-warping (M) methods, three methods of time-domain transformation, including permutation (P), time-warping, and cropping methods, and four methods featuring mixtures of the aforementioned methods, which are R & S, R & J, J & P, and R & S & M. The evaluated performances are reported based on the indices, which are the F1-score, accuracy, recall, and precision. The baseline of performance is also evaluated, without featuring any data augmentation methods.

For mild PD classification using deep learning based on IMU-derived gait data, the superior data-augmentation method was investigated. Eleven different methods for data augmentation were considered, as illustrated in Fig. 3. Mild PD classification results obtained from representative CNN-based classification methods were compared with the IMU-collected gait data to which various data extensions were applied, and performances of these data extension methods were accordingly ranked. For evaluating the classification of these data augmentation methods, the F1-score, accuracy, recall, and precision were used (fivefold cross validation). The evaluation results are presented in Table 1. To visualize the resulting performance, Fig. 3 is presented for reference. Observably, the rotation method for data augmentation yielded the best classification performance in terms of the F1-score, accuracy, and recall. In terms of only precision, the scaling method for data augmentation exhibited the best classification performance. All the evaluation scores suggested that the method using the magnitude-domain transformation exhibited the best performance compared with that using the time-domain transformation.

**Table 1:** Performance-evaluation results for data augmentation methods.

Data augmentation method	F1-score (%)	Accuracy (%)	Recall (%)	Precision (%)
Nothing	83.2	81.9	87.1	78.5
Magnitude-domain transformation				
Jittering (J)	87.5	83.8	91.0	84.6
Magnitude warping (M)	85.9	81.5	90.0	82.2
Rotation (R)	88.1	86.4	91.7	84.8
Scaling (S)	87.5	84.5	90.8	85.2
Time-domain transformation				
Time warping	85.5	81.6	90.3	82.2
Permutation (P)	84.7	81.1	88.4	81.7
Cropping	86.9	83.8	90.9	85.1
Mix data augmentation				
R & S	86.3	85.5	86.6	88
R & J	84.4	81.2	86.7	81.7
J & P	86.1	82.4	89.1	83.8
R & S & M	86.7	82.8	89.7	84.4

The data augmentation methods are as follows: Four methods of magnitude-domain transformation, including rotation (R), jittering (J), scaling (S), and magnitude-warping (M) methods, three methods of time-domain transformation, including permutation (P), time-warping, and cropping methods, and four methods featuring mixtures of the aforementioned methods, which are R & S, R & J, J & P, and R & S & M. The evaluated performances are reported based on the indices, which are the F1-score, accuracy, recall, and precision. The baseline of performance is also evaluated, without featuring any data augmentation methods.

As supplemental investigation, mixtures of data augmentation methods featuring a combination of these transformations were also used for additional evaluation. This study experimentally examined the following four types of mixed data-augmentation methods. The first was the combination of rotation and scaling, which combined the rotation with the highest scores in the F1-score, accuracy, and recall and the scaling with the highest score in the precision. The second was the combination of rotation and jittering, which combined the rotation and jittering with the highest scores in recall, where these two values were the highest among all evaluation scores. The third was the combination of jittering and permutation, which combined two representative methods that were not the highest in the evaluation scores in each magnitude-domain transformation and the time-domain transformation in the data augmentation. The fourth was the combinations of rotation, scaling, and magnitude warping, which combined the three representative methods in the magnitude-domain transformation.

Including the results of this supplementary investigation, the results indicated that the rotation method, which is a mixed data-augmentation mode featuring the rotation and scaling methods, rendered the best performance in terms of precision. Furthermore, the method with the best performance was related to the magnitude-domain transformation, specifically including the rotation method. In particular, in the experimental results for mild PD classification using deep learning based on IMU-recorded gait data, the rotation method emerged as the superior data-augmentation method.

## Discussion

Several data augmentation techniques were identified and explored in this study, and effective data augmentation methods were investigated for the classification of mild PD cases and HPs through deep-learning. The experimental results demonstrated that CNN-based methods performed superiorly in terms of the F1-score and classification accuracy when the rotation method was applied as the data augmentation method. This study exhaustively investigated and compared various data augmentation methods, revealing that the rotation method was the appropriate data-augmentation method in the mild PD-classification framework based on IMU-recorded gait data. This result is based on the dataset solely generated with sensor data collected from the left and right shank-worn IMUs; this is a pioneering work yielding a low-cost and simple approach for gait analysis using an IMU sensor.

The experimental results revealed that among the magnitude-domain transformation-based data augmentations, the rotation method maximally improved the classification performance. The rotation method is a random coordinate transformation of the IMU-sensor data regarding the x-, y-, and z-axes accelerations, which augments the data. The range of random angle parameters utilized in the coordinate transformation was ± 15°. This data augmentation method corresponded well with the postural changes of the foot during gait. Considering the biomedical features of gait, the posture changes of the foot were reported based on the foot-contact angle, which was formed between an axis connecting the toe-heel relative to the ground reference in the sagittal plane at heel contact and the toe-out angle, which was defined at heel contact in the transverse plane as the toe-heel line relative to the walking (anterior) direction. In a study, healthy young individuals exhibited foot-contact and toe-out angles of 20° and 12°, respectively^32^, implying that the postural change of the foot was approximately ± 15°. Studies have suggested that appropriate data-augmentation methods should be selected by considering the data characteristics^20^. In this regard, the rotation method was considered the suitable data-augmentation method for reflecting gait mechanics.

The magnitude-domain transformation, time-domain transformation, and mixed data-augmentation featuring these two methods in various data augmentation methods were comparatively studied. Interestingly, the magnitude-domain transformation yielded the best result in terms of classification performance. As important evaluation indexes in the magnitude-domain transformation, the accuracy and lower and upper limits of the F1-score range tended to be greater than those in the other methods. In the evaluation indexes of classification performance, the following ranges of the F1-score were observed with each data augmentation method: 85.9–88.1 for magnitude-domain transformation, 84.7–86.9 for time-domain transformation, and 84.4–86.7 for mixed data-augmentation, compared with a score of 83.2 for the baseline (with no data augmentation). Similarly, the following ranges of the accuracy were achieved with each data augmentation method for the classification: 81.5–86.4 for magnitude-domain transformation, 81.1–83.8 for time-domain transformation, and 81.2–85.5 for mixed data-augmentation, compared with a score of 81.9 for the baseline (with no data augmentation). Thus, these results suggest that for detecting mild PD patients compared with elderly healthy people, magnitude-domain transformation greatly enhances the performance of deep learning classification.

The effectiveness of the magnitude-domain transformation effectiveness, compared with those of the other approaches, is considered an important aspect. Notably, revealing this aspect is crucial for maintaining the data order as a time-series structure and for modifying the data. In data augmentation through magnitude-domain transformation, the values of each element are modified, and the time steps are maintained constant. In contrast, in augmentation via time-domain transformation, the elements of the time series are displaced to time steps that differ from the original sequence. Because human gait is a dynamic system, the time-series associated with the temporal evolution of measured data is considered a prime characteristic of gait.

The time-domain transformation and mixed data-augmentation did not perform optimally, this trend differs significantly from that reported in a previous work. Um et al.^20^ reported that for Parkinson’s disease monitoring using CNN, the mixed data-augmentation method, comprising the rotation, permutation, and time-warping methods, exhibited the best performance for acceleration data obtained via wrist-worn sensors. Although the contribution of rotation to high performance was similar, the contributions of permutation and time warping methods differed from those reported in this study. The wrist-acceleration data used in the previous study featured a greater number of static characteristics, rather than periodic information concerning gait, and the replacement of subintervals in the permutation method and addition of a dynamic bias over time in the time-warping method may have been effective, unlike that observed in the present study. Additionally, Iwana et al.^21^ discovered that the rotation, permutation, and time-warping methods experienced significantly degraded accuracies for the classification of 128 datasets including 9 devices. In contrast, our research revealed a positive result, that is, rotation had severely detrimental effects on accuracy. This result indicates that different types of datasets possess different properties, and an efficient dataset must be determined for PD diagnosis.

In this study, the contribution of magnitude-domain transformation toward performance greatly outweighed those of the time-domain transformation and mixed-data augmentation. The magnitude-domain transformations moderately enhanced the accuracy; these similar transformations tend to act uniformly but only differ in terms of the number of directions in which the magnitude is scaled. Considering their positive effect as data augmentation methods, magnitude-domain transformations emerge as a prime candidate for gait classification, especially for mild-PD diagnosis, for time-series data augmentation. In contrast, the time-domain transformations did not perform optimally for the classification task. Presumably, the time-series were over-transformed, causing significant noise and distorting the time characteristics. Similarly, the permutation method did not perform well: this method disrupted the time dependency of the time-series. Generally, permutation should be used for periodic or extremely sparse time-series. Observably, the time-domain transformations did not perform adequately. In the mixed data-augmentation method, the data are transformed twice based on individual methods. Notably, the combination of augmentation methods did not perform optimally compared with the individual method; the multiple data-augmentation method distorted the data severely, and thus, the feature may not be readily recognized by the model.

In this field, this study is a pioneering application of data augmentation for classifying mild PD and healthy elderly individuals, especially using a small number of IMU sensors (one IMU sensor), yielding a relatively high accuracy. In PD-related research, for PD analysis via IMU sensors, Camps^33^ proposed a system through deep learning for freezing of gait detection in patients with PD at their residences using a single waist-worn IMU. Moreover, for PD state assessment, assist systems^34,35^ have been used to classify the PD state as asleep, off, on, and (troublesome) dyskinesia for motor-symptom monitoring. In another IMU sensor-related study, Dehzangi et al. devised a deep CNN network with early and late sensor fusion approaches to identify 10 subjects with 5 IMU sensors attached at the chest, lower back, right-hand wrist, right knee, and right ankle^14^. Nguyen et al. proposed an IMU-based spectrogram approach with CNN for gait classification of abnormal gait in musculoskeletal disease^15^. In contrast to these studies, the present study specifically explored the classification of mild PD and healthy subjects based on IMU gait data. The results of this study are expected to contribute to future studies on the classification of patients with mild PD and healthy individuals.

This study focused on the classification of mild Parkinson's disease and healthy subjects based on the minute differences in their gait movements. As a first step, this study reported a comparison of the effectiveness of relatively traditional methods for data augmentation. The visual differentiation between mild Parkinson's disease and normal subjects' gait is difficult even for medical specialists and is considered one of the most important issues to address. However, a previous study reported that using generative adversarial networks (GANs) to augmented data from IMU sensors attached to the entire human body can improve the classification accuracy of motor actions that can be visually discriminated by human eyes^36,37^. Therefore, data augmentation using GANs may also be effective in improving the classification accuracy of fine differences in walking movements between mild Parkinson's disease and healthy subjects. Investigating this effectiveness is a future issue.

This study investigated the challenging task of visually classifying PD and healthy gait based on ankle-mounted IMU-sensor data. PD gait is considered as one of the abnormal gait types. In a previous study, Nguyen et al. proposed an IMU-based spectrogram approach and a CNN-based gait classification method to detect abnormal gait corresponding to musculoskeletal disorders. The gait spectrogram dataset was generated using time–frequency analysis^15^. Building on this prior work, we opted to train our model using the results of time–frequency analysis as a first step. However, for classification models that utilize deep learning, methods that directly train models on raw data from IMU sensors have potential to be effective. Exploring this direction is one of our future tasks.

In a preliminary experiment, this study compared classification accuracies based on augmentation factors of + 100%, + 200%, and + 300% using the rotation method for data augmentation on the same IMU dataset. The results indicated that the + 100% augmentation factor performed the best. Therefore, for comparing data augmentation methods in this study, we fixed the augmentation factor for training data at + 100%. However, the dependence of the augmentation coefficient requires further investigation.

This study used CNN, the most basic deep learning model, and compared it with various data augmentation methods. However, exploring the latest deep learning models, such as RNN and Transformer, is necessary for future research and represents a future task.

## Conclusion

For the classification of mild-PD patients and healthy elderly participants, based on time-series data obtained via a shank-worn IMU, this study investigated effective data augmentation methods for a representative deep learning model. In summary, several data augmentation methods were applied to the representative CNN model, and their classification performances for PD-patient detection were compared. As a representative model of deep learning, a CNN comprising four 2D convolutional layers was constructed and applied for the performance evaluation of the data augmentations. Overall, 11 different data augmentation methods were applied to this representative CNN model, and their performances were compared for identifying the most appropriate data augmentation method for time-series data. The data augmentation methods were four methods of magnitude-domain transformation, including rotation, jittering, scaling, and magnitude warping methods, three methods of time-domain transformation, including permutation, time warping, and cropping methods, and four methods of mixed data-augmentation methods, featuring these two types of augmentation. As revealed by the evaluation results, compared to the methods of time-domain transformation and mixed methods, the methods of magnitude-domain transformation were more advantageous for improving classification performance. Especially, compared to the baseline performance (no data augmentation) in terms of the F1-score (81.9%) and accuracy (83.2%), the rotation method in the magnitude-domain transformation yielded the best performance in terms of the F1-score (88.1%) and accuracy (86.4%), *i*.*e*. the F1-score and accuracy were improved by 5.5% and 5.9%, respectively. The rotation method was a random coordinate transformation of the IMU sensor's data depicting the x-, y-, and z-axes accelerations, which augmented the data. Presumably, this data augmentation method corresponded well with the postural changes of the foot during gait. The effectiveness of the magnitude-domain transformation, compared with those of the other approaches, was considered important. Notably, revealing this aspect was essential for maintaining the data order as a time-series structure and for modifying the data. The selection of such appropriate data extensions would further contribute toward improving the classification performance for mild PD detection.

## Data Availability

The datasets generated and/or analyzed during the current study are available from the corresponding author on reasonable request.
